# Case Report: Unicentric Castleman disease at the superior border of the pancreatic body mimicking a pancreatic neuroendocrine tumor: a case report and literature review

**DOI:** 10.3389/fsurg.2026.1947071

**Published:** 2026-09-11

**Authors:** Linru Yang, Jin Liu, Junhua Zhang

**Affiliations:** 1 First Clinical Medical College, Gansu University of Chinese Medicine, Lanzhou, China; 2 Department of Surgical Oncology, Gansu Provincial Hospital, Lanzhou, China; 3 Department of Critical Care Medicine, Lanzhou Petrochemical General Hospital (The Fourth Affiliated Hospital of Gansu University of Chinese Medicine), Lanzhou, China

**Keywords:** case report, differential diagnosis, laparoscopic excision, pancreatic neuroendocrine tumor, peripancreatic mass, unicentric Castleman disease

## Abstract

Peripancreatic unicentric Castleman disease is rare and may mimic a primary pancreatic neoplasm because it often presents as a well-circumscribed hypervascular mass. We report a 26-year-old woman with type 2 diabetes mellitus in whom an incidentally detected upper abdominal mass was initially considered to arise from the pancreatic body. Computed tomography and magnetic resonance imaging showed a sharply marginated hypervascular lesion with diffusion restriction at the superior border of the pancreatic body, leading to a preoperative suspicion of pancreatic neuroendocrine tumor. Laparoscopic exploration revealed a well-encapsulated mass in the lesser sac closely abutting the superior surface of the pancreas. A clear dissection plane between the lesion and the pancreatic parenchyma allowed complete excision without pancreatic resection or splenectomy. Histopathological examination confirmed hyaline vascular-type unicentric Castleman disease. The postoperative course was uneventful, and no residual or recurrent lesion was detected during 1 year of follow-up; glycemic status also remained stable. This case highlights that close proximity of a hypervascular mass to the pancreas does not necessarily indicate pancreatic origin. Careful multiplanar imaging and direct intraoperative assessment of the lesion–pancreas interface may help determine the appropriate surgical extent and avoid unnecessary pancreatic resection when complete excision is technically feasible.

## Introduction

Castleman disease (CD) is an uncommon lymphoproliferative disorder characterized by benign lymph node hyperplasia (1–3). It most often involves the mediastinum, neck, abdomen, or retroperitoneum, whereas pancreatic and peripancreatic presentations are rare (2). Preoperative diagnosis can be difficult because symptoms, laboratory findings, and serum tumor markers are usually nonspecific. When CD develops near the pancreas, it may appear as a well-circumscribed hypervascular mass and closely resemble a pancreatic neuroendocrine tumor (pNET), particularly when arterial enhancement and diffusion restriction are present (4–6).

An additional diagnostic challenge is determining whether a mass truly arises from the pancreatic parenchyma or is located adjacent to the gland. This distinction directly influences operative planning because a presumed pancreatic neoplasm may lead to distal pancreatectomy, pancreaticoduodenectomy, or splenectomy, whereas an encapsulated peripancreatic lesion may be amenable to complete local excision. We report a young woman with hyaline vascular-type CD located at the superior border of the pancreatic body and initially suspected to have a pNET. The lesion was completely excised laparoscopically after a clear plane from the pancreatic surface was confirmed. A review of previously reported pancreatic and peripancreatic cases is also presented.

## Case presentation

A 26-year-old woman was admitted to Gansu Provincial Hospital for further evaluation of an upper abdominal mass that had been detected incidentally during a routine health examination in May 2025. She was in good general condition and reported no abdominal pain, fever, jaundice, nausea, vomiting, weight loss, or other constitutional symptoms.

The patient was 166 cm tall and weighed 50 kg, corresponding to a body mass index of 18.1 kg/m^2^. She had been diagnosed with type 2 diabetes mellitus 2 years earlier and was receiving metformin without insulin therapy. She reported occasional alcohol consumption and denied smoking. There was no history of pancreatitis, peptic ulcer disease, gastrointestinal surgery, or malignant disease, and her family history was negative for malignancy.

An outside contrast-enhanced computed tomography (CT) examination had identified an enhancing mass adjacent to the pancreatic body, and pNET was considered the leading preoperative diagnosis. She was therefore referred to our hospital for further evaluation and surgical management.

Physical examination was unremarkable. Complete blood count, liver and renal function tests, serum electrolytes, coagulation profile, and pancreatic enzymes showed no clinically significant abnormalities. Glycated hemoglobin was 6.12% (institutional reference range, 4.0%–6.0%). Serum tumor markers were within the reference ranges: carcinoembryonic antigen, 1.3 ng/mL (reference, <5 ng/mL); carbohydrate antigen 125, 9.4 U/mL (reference, <35 U/mL); carbohydrate antigen 19-9, 10.50 U/mL (reference, <37 U/mL); and carbohydrate antigen 242, 11.16 U/mL (reference, <25 U/mL).

**Figure 1:**
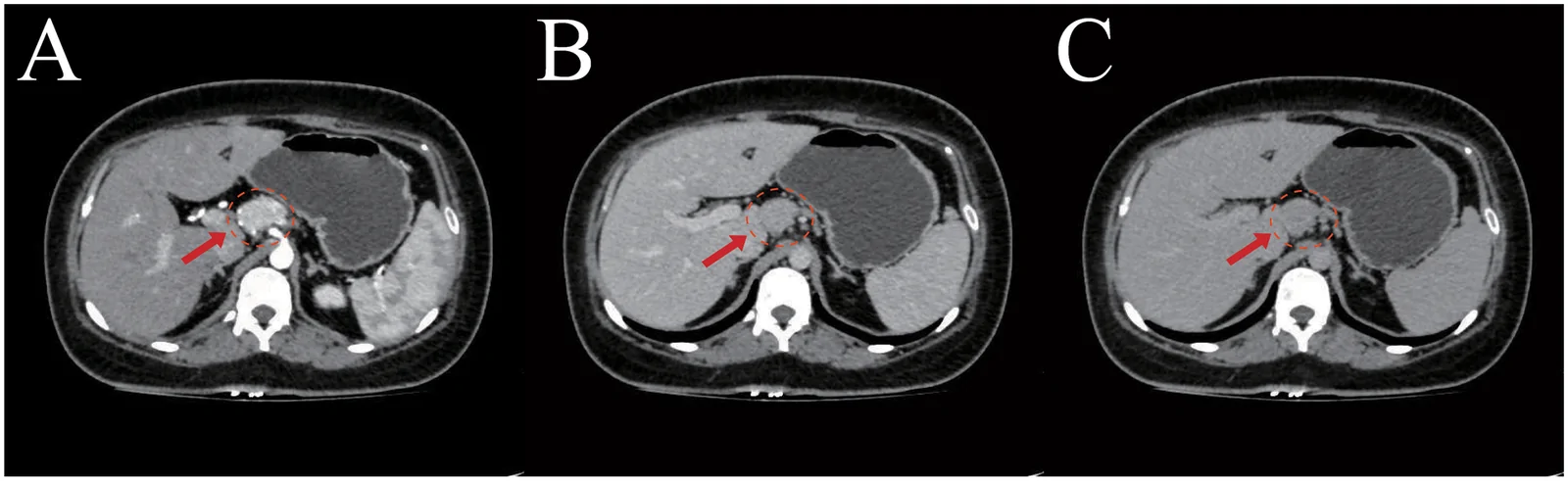
Contrast-enhanced abdominal CT findings. **(A)** Arterial phase showing a round, well-defined hypervascular mass at the superior border of the pancreatic body (mean attenuation, 146.7 HU). **(B)** Portal venous phase showing persistent but lower enhancement (mean attenuation, 96.3 HU). **(C)** Delayed phase showing further decreased attenuation (mean attenuation, 75.3 HU). Red arrows and dashed circles indicate the lesion.

Contrast-enhanced CT showed a round, sharply marginated soft-tissue mass measuring approximately 2.6 × 3.4 cm at the superior border of the pancreatic body and projecting toward the hepatogastric space (Figure 1). Quantitative region-of-interest measurements showed mean attenuation values of 146.7 HU in the arterial phase, 96.3 HU in the portal venous phase, and 75.3 HU in the delayed phase, demonstrating prominent arterial enhancement with decreasing attenuation on the later phases. The margin was smooth, and no invasive features were identified. No additional abdominal or retroperitoneal lymphadenopathy was identified on the available CT and MRI examinations.

**Figure 2:**
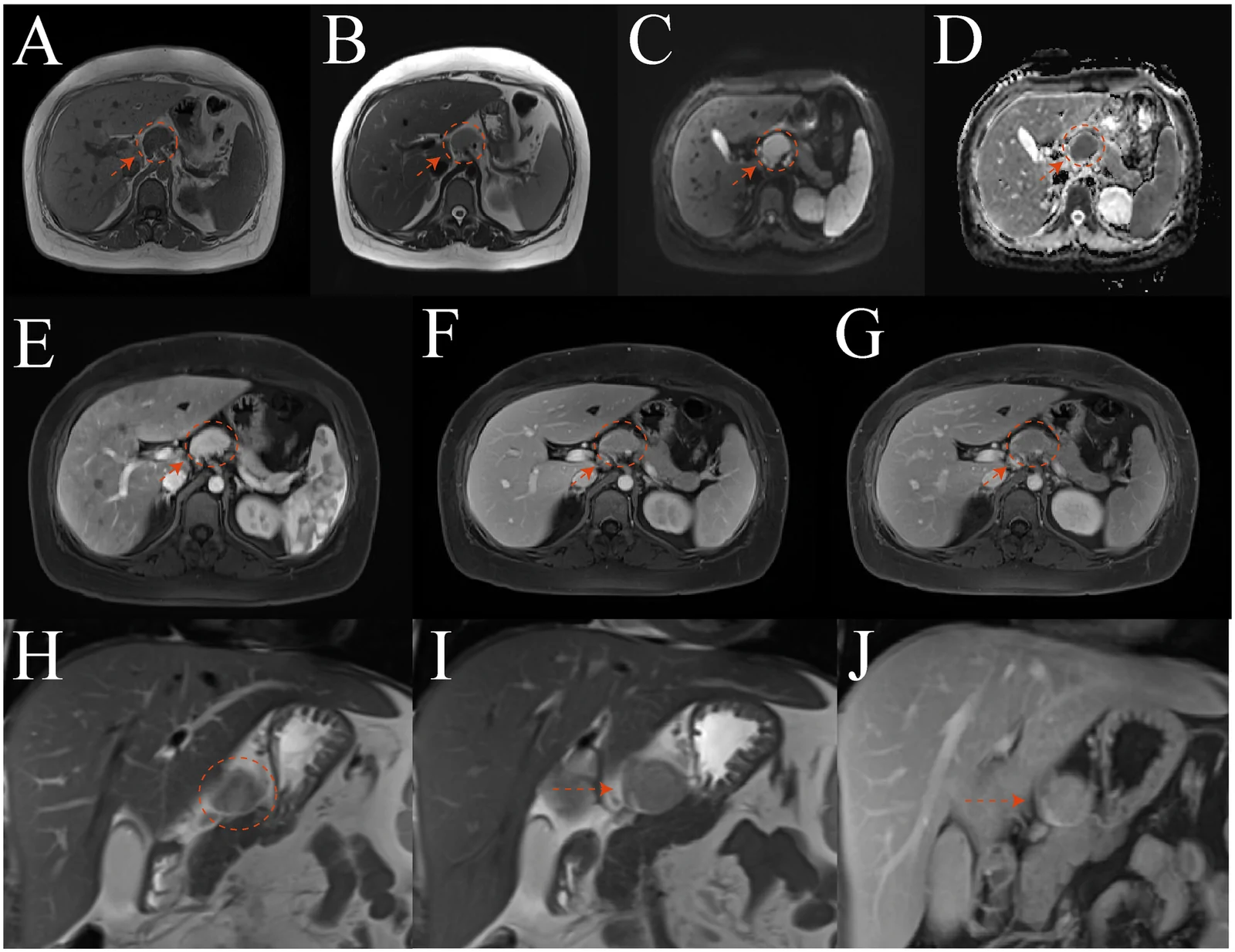
Magnetic resonance imaging findings of the peripancreatic mass. The lesion was hypointense on axial T1-weighted imaging **(A)** and mildly hyperintense on axial T2-weighted imaging **(B)** Diffusion-weighted imaging showed hyperintensity **(C)**, with corresponding hypointensity on the apparent diffusion coefficient map **(D)**; the mean ADC value was approximately 0.73 × 10⁻^3^ mm^2^/s, consistent with restricted diffusion. Dynamic contrast-enhanced MRI demonstrated marked arterial-phase enhancement **(E)**, followed by relatively lower enhancement during the portal venous **(F)** and delayed phases **(G)** Coronal T2-weighted images from adjacent sections **(H,I)** localized the lesion to the superior border of the pancreatic body. On retrospective review, a subtle intervening tissue plane between part of the lesion and the pancreatic surface was identified (arrow in I), although a complete radiological capsule could not be confidently identified. The delayed-phase contrast-enhanced coronal image **(J)** further demonstrated close apposition of the lesion to the pancreatic surface without convincing evidence of intrapancreatic origin. Red dashed circles and arrows identify the lesion; in panel I, the arrow specifically indicates the lesion–pancreas interface.

Upper abdominal magnetic resonance imaging (MRI) revealed a well-circumscribed mass measuring approximately 4.3 × 2.9 × 3.6 cm. The lesion was hypointense on axial T1-weighted imaging and mildly hyperintense on T2-weighted imaging (Figures 2A, B). Diffusion-weighted imaging demonstrated hyperintensity with corresponding hypointensity on the apparent diffusion coefficient map, with a mean ADC value of approximately 0.73 × 10⁻^3^ mm^2^/s, consistent with restricted diffusion (Figures 2C, D). Dynamic contrast-enhanced MRI showed marked arterial-phase enhancement, followed by relatively lower enhancement during the portal venous and delayed phases (Figure 2E–G). Retrospective review of the coronal T2-weighted images demonstrated a subtle intervening tissue plane between part of the lesion and the superior surface of the pancreas, supporting an extrapancreatic location. However, a complete and definite radiological capsule could not be confidently identified. The delayed-phase contrast-enhanced coronal image further demonstrated close apposition of the lesion to the pancreatic surface without convincing evidence of intrapancreatic origin (Figure 2H–J). The imaging appearance favored pNET or a neurogenic tumor rather than conventional pancreatic ductal adenocarcinoma, but a definitive diagnosis could not be established. Because the mass was localized, well circumscribed, and radiologically resectable, a laparoscopic approach was selected to permit direct assessment of its relationship with the pancreas before determining the extent of surgery.

Laparoscopic exploration revealed a soft, well-encapsulated mass in the lesser sac at the superior surface of the pancreatic body. The mass closely abutted the gland but had a clear dissection plane. After the lesser sac was opened with an ultrasonic scalpel, the lesion was carefully separated from the pancreatic surface and completely excised without distal pancreatectomy, pancreaticoduodenectomy, or splenectomy. The gross surgical specimen, including the encapsulated lesion and a small amount of attached adipose tissue, measured approximately 6 × 3 × 2 cm.

**Figure 3:**
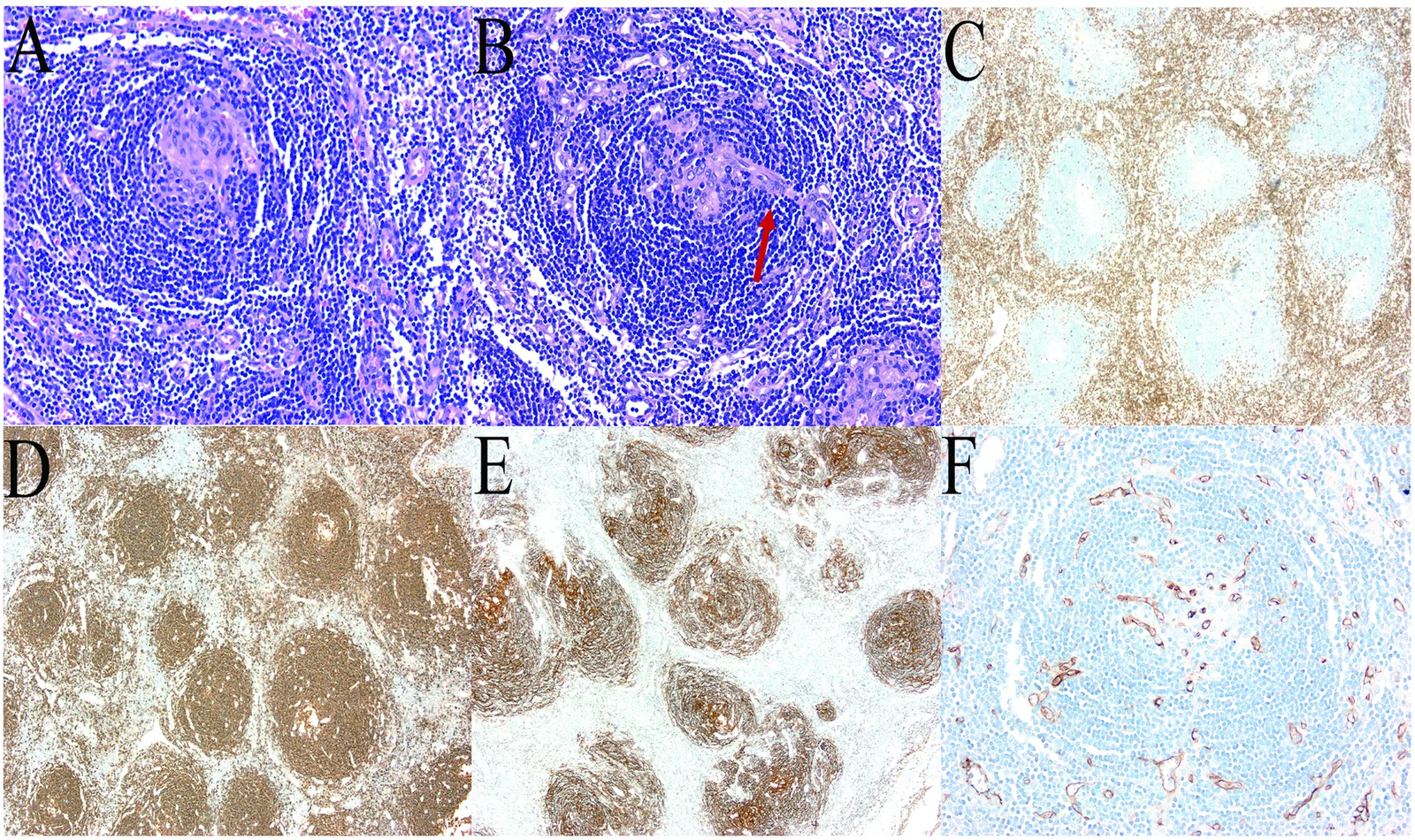
Histopathological and immunohistochemical findings. **(A,B)** Hematoxylin and eosin staining showed lymphoid follicular hyperplasia with hyalinized vessels penetrating the germinal centers and concentric mantle-zone lymphocytes, forming characteristic onion-skin-like and lollipop-like structures (original magnification, ×200). **(C)** CD3-positive T cells were mainly distributed in interfollicular areas. **(D)** CD20-positive B cells were predominantly present within lymphoid follicles. **(E)** CD21 highlighted preserved follicular dendritic cell meshworks. **(F)** CD34 demonstrated abundant vascular proliferation.

Gross examination showed a gray-yellow nodular soft-tissue mass with a visible capsule. The differences among the reported CT, MRI, and gross dimensions likely reflect differences in imaging planes and measurement methods; additionally, the gross measurement represented the entire resected specimen, including the encapsulated lesion and attached adipose tissue, rather than the lesion alone. Histopathological examination demonstrated prominent lymphoid follicles with expanded mantle zones. Small hyalinized vessels penetrated the germinal centers, and the mantle-zone lymphocytes were arranged concentrically, producing characteristic onion-skin-like and lollipop-like appearances (Figures 3A, B).

Immunohistochemical findings supported the diagnosis. CD3-positive T cells were mainly distributed in the interfollicular areas, whereas CD20-positive B cells were predominantly present within lymphoid follicles. CD21 highlighted preserved follicular dendritic cell meshworks, and CD34 demonstrated abundant vascular proliferation (Figure 3C–F). CD30 staining and Epstein–Barr virus-encoded RNA *in situ* hybridization were negative. Ki-67 labeling was high within lymphoid follicles and lower in the interfollicular areas. In the context of a solitary lesion, absence of constitutional symptoms, and no additional lymphadenopathy on the available imaging, the final pathological diagnosis was hyaline vascular-type unicentric Castleman disease.

**Figure 4:**
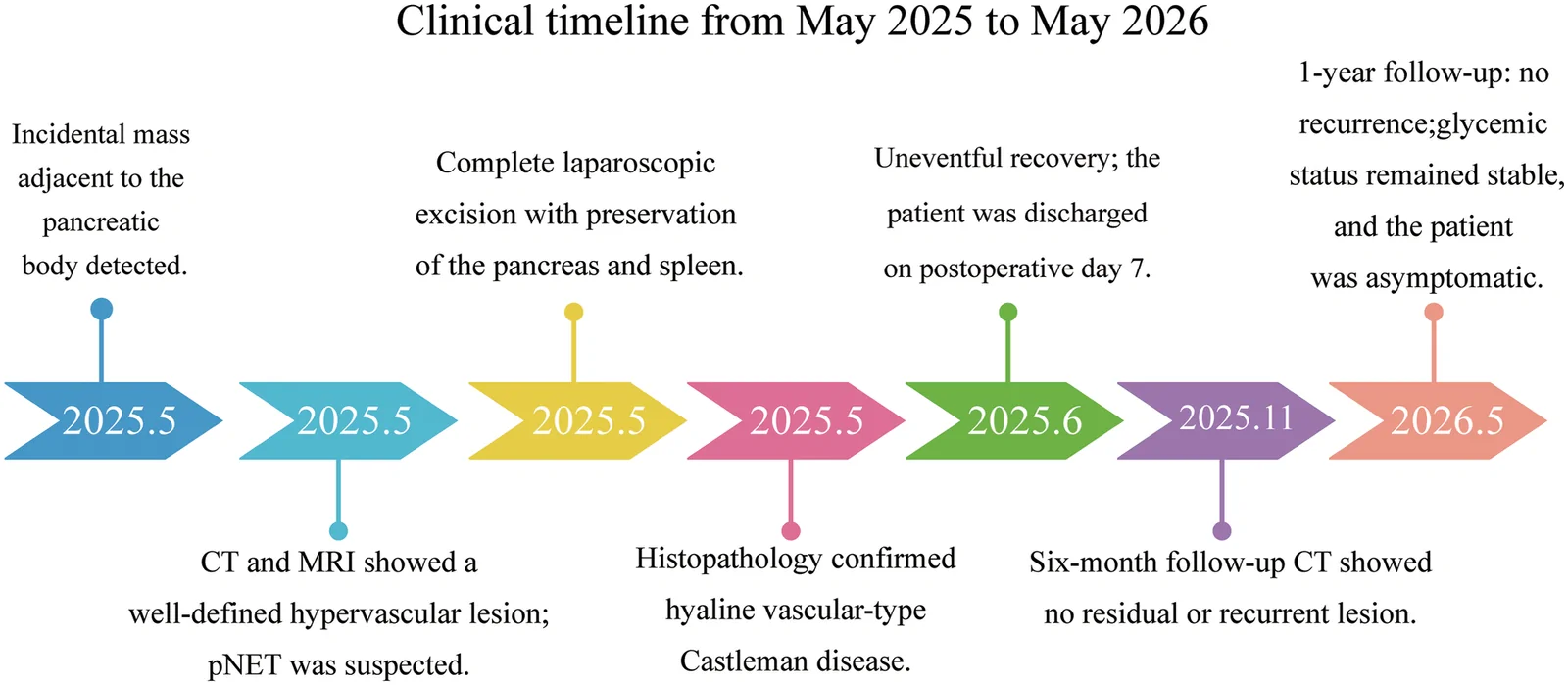
Clinical timeline summarizing detection of the lesion, preoperative assessment, laparoscopic excision, pathological confirmation, and follow-up from May 2025 to May 2026.

The postoperative course was uneventful, and the patient was discharged on postoperative day 7. At 1 month, abdominal and retroperitoneal ultrasonography showed normal pancreatic morphology without a residual mass, and glycemic control remained stable. Follow-up CT at 6 months showed no residual or recurrent lesion. At the most recent follow-up in May 2026, repeat CT and blood glucose assessment remained unremarkable. The patient was asymptomatic and reported satisfaction with the treatment outcome. The clinical timeline is summarized in Figure 4. This report was prepared in accordance with the CARE guidelines.

## Discussion

The central diagnostic issue in this case was not the histological diagnosis of CD, which was supported by characteristic architecture and immunophenotype, but the anatomical origin of the mass. Preoperative axial imaging initially suggested a pancreatic body lesion. However, retrospective review of the coronal images and the operative finding of a clear plane from the pancreatic surface support the more accurate description of a peripancreatic mass at the superior border of the pancreatic body. Close apposition to the gland should not be equated with origin from pancreatic parenchyma. Although a complete capsule was not definitively visible on preoperative imaging, the subtle intervening tissue plane identified on coronal MRI was consistent with the clear dissection plane observed intraoperatively, while gross examination subsequently confirmed a visible capsule. The solitary presentation, absence of constitutional symptoms, and lack of additional lymphadenopathy on the available imaging supported classification as unicentric CD.

To place this presentation in context, we reviewed 17 previously reported cases of CD in the pancreatic or peripancreatic region published between 2015 and 2026 (Table 1) (7–23). Most lesions were initially considered to be pNET, pancreatic malignancy, or an indeterminate pancreatic tumor. The anatomical terminology in these reports was heterogeneous and included pancreatic, peripancreatic, retroperitoneal, and hepatopancreatic locations. A previous report described unicentric CD as a retroperitoneal mass at the upper edge of the pancreas (13). Collectively, these cases show that a hypervascular lymphoid mass adjacent to the pancreas can enter the diagnostic pathway of primary pancreatic neoplasia even when its true origin is extra-pancreatic.

**Table 1:** Previously reported cases of castleman disease in the pancreatic or peripancreatic region.

First author, year	Age/sex	Presentation	Work-up	Preoperative impression	Treatment	Ref.
Matsumoto, 2015	74/M	Asymptomatic	CT, EUS	Pancreatic malignancy	PD	(7)
Abdessayed, 2017	34/F	Abdominal discomfort	US, MRI, CT	pNET	Open surgery	(8)
Ozsoy, 2018	62/F	Epigastric discomfort	CT, PET/CT	No definite diagnosis	PD	(9)
Ferreira Junior, 2019	34/F	Abdominal pain	MRI	Pancreatic malignancy	Open surgery	(10)
Gülmez, 2019	38/F	Abdominal pain	CT, PET/CT	Pancreatic tumor	Laparoscopic resection	(11)
Huang, 2020	44/M	Asymptomatic	MRI	No definite diagnosis	PD	(12)
Zhou, 2020	33/F	Asymptomatic	MRI, CT	No definite diagnosis	Open surgery	(13)
Gunda, 2021	58/F	Asymptomatic	CT, MRI, PET/CT	pNET	DP + splenectomy	(14)
Zhai, 2022	44/F	Asymptomatic	CEUS, MRI, CT	pNET	Open surgery	(15)
Liu, 2022	28/F	Asymptomatic	CT, PET/CT	Pancreatic malignancy	Open surgery	(16)
Gou, 2023	46/M	Upper abdominal pain	CT, EUS-FNA	pNET	Open surgery	(17)
Dev, 2023	46/F	Asymptomatic	CT	pNET	RAMPS	(18)
Jin, 2024	26/F	Asymptomatic	CEUS, CT, MRI, PET/CT	SPT or lymphatic origin	Laparoscopic excision	(19)
Pham, 2025	53/M	Asymptomatic	US, CT	Pancreatic malignancy	Open surgery	(20)
Xu, 2025	36/M	Asymptomatic	US, CT	pNET	Open surgery	(21)
Solanki, 2026	34/F	Vague abdominal pain	US, CT, EUS-FNB, FDG-PET	pNET or GIST	Open mass excision	(22)
Matsui, 2026	55/M	Epigastric pain	CT, MRI, EUS-FNA	pNET or lymphoma	RAMPS	(23)

CEUS, contrast-enhanced ultrasound; CT, computed tomography; DP, distal pancreatectomy; EUS, endoscopic ultrasound; EUS-FNA, endoscopic ultrasound-guided fine-needle aspiration; EUS-FNB, endoscopic ultrasound-guided fine-needle biopsy; F, female; FDG-PET, fluorodeoxyglucose positron emission tomography; GIST, gastrointestinal stromal tumor; M, male; MRI, magnetic resonance imaging; PD, pancreaticoduodenectomy; PET/CT, positron emission tomography/computed tomography; pNET, pancreatic neuroendocrine tumor; RAMPS, radical antegrade modular pancreatosplenectomy; SPT, solid pseudopapillary tumor; US, ultrasound.

The imaging findings in our patient explain the preoperative suspicion of pNET. Marked arterial enhancement and diffusion restriction are well-recognized features of pNET, but they are not specific (4–6). CD, paraganglioma, solid pseudopapillary tumor, accessory splenic tissue, and other hypervascular lesions may show overlapping appearances. For masses near the pancreas, evaluation should therefore extend beyond enhancement characteristics. Multiplanar images should be used to assess the center of the lesion, the presence of a preserved fat or tissue plane, continuity with pancreatic parenchyma, pancreatic contour, and the relationship to the pancreatic duct and adjacent vessels. In this case, the coronal images clarified the superior-border location, while the definitive anatomical relationship was established during laparoscopy.

Preoperative tissue sampling may be useful in selected patients, but its limitations should be recognized. The reviewed cases included contrast-enhanced ultrasound, CT, MRI, positron emission tomography/CT, endoscopic ultrasound, and endoscopic ultrasound-guided fine-needle aspiration or biopsy. Nevertheless, the diagnosis was generally established after surgical excision. In one reported case, fine-needle aspiration suggested pNET, whereas the resection specimen confirmed hyaline vascular-type CD (17). Limited cytological or biopsy material may contain lymphoid cells or vascular fragments but fail to preserve the follicular architecture required for a confident diagnosis. A nondiagnostic or apparently neuroendocrine biopsy result should therefore be interpreted cautiously when CD remains within the differential diagnosis.

Histopathology remains the diagnostic standard. Typical features of the hyaline vascular type include regressed or hyalinized germinal centers, penetrating vessels, expanded mantle zones arranged in an onion-skin pattern, and prominent interfollicular vascular proliferation (3). The present lesion demonstrated follicular hyperplasia, lollipop-like vascular penetration, onion-skin-like mantle-zone arrangement, a CD21-positive follicular dendritic cell network, and CD34-highlighted vascular proliferation. Negative CD30 staining and Epstein–Barr virus-encoded RNA *in situ* hybridization did not support a CD30-positive or EBV-associated lymphoproliferative process.

The extent of surgery is the principal management concern when a hypervascular mass is believed to be pancreatic. As shown in Table 1, reported patients underwent procedures ranging from local excision to pancreaticoduodenectomy, distal pancreatectomy with splenectomy, and radical antegrade modular pancreatosplenectomy (7–23). This variation probably reflects both diagnostic uncertainty and difficulty determining the lesion–pancreas interface. Complete surgical excision is the preferred treatment for resectable unicentric CD (2), but complete excision does not require formal pancreatic resection when the lesion is clearly extra-parenchymal and technically separable.

In our patient, the decision to avoid pancreatic resection was based primarily on direct confirmation of a safe dissection plane, not merely on her age, low body mass index, or diabetes. These patient factors strengthened the desirability of preserving pancreatic tissue after technical safety had been established. Distal pancreatectomy and larger resections may be followed by new-onset or worsened endocrine dysfunction (24, 25). Thus, the operative strategy balanced diagnostic uncertainty, completeness of excision, avoidance of unnecessary morbidity, and preservation of pancreatic endocrine reserve.

This case should not be interpreted as supporting local excision for every hypervascular lesion near the pancreas. Formal pancreatic resection may still be required when a lesion is inseparable from the gland, demonstrates invasive features, involves the pancreatic duct or major vessels, or remains highly suspicious for malignancy after appropriate assessment. Conversely, when laparoscopy demonstrates a well-encapsulated mass with a clear plane from the pancreatic surface, complete excision without pancreatic resection may provide both diagnosis and treatment. In the present case, this approach was followed by uncomplicated recovery, stable glycemic control, and no residual or recurrent disease during 1 year of follow-up.

This report has several limitations. First, CD was not diagnosed before surgery, reflecting the nonspecific imaging appearance and the rarity of this location. Second, the literature review was narrative and intended to provide clinical context rather than serve as a systematic review. Terminology and anatomical descriptions varied among the included reports, and the precise relationship between each lesion and pancreatic parenchyma could not be independently verified. Some lesions reported as pancreatic CD may therefore have been peripancreatic or retroperitoneal masses closely adjacent to the gland. Third, the available coronal images improved localization but did not by themselves prove the lesion's origin; the final designation relied on the combined imaging and operative findings. Finally, longer follow-up is needed to confirm the durability of the outcome.

## Conclusion

Castleman disease located at the superior border of the pancreatic body can closely mimic a pNET because of its sharply marginated and hypervascular appearance. Accurate terminology should reflect the demonstrated anatomical relationship rather than assume pancreatic origin. Multiplanar imaging and intraoperative assessment of the lesion–pancreas interface are important for surgical planning. When a localized lesion can be safely separated from the pancreatic surface, complete laparoscopic excision without pancreatic resection may establish the diagnosis and treat the disease while avoiding unnecessary loss of pancreatic parenchyma.

## Data Availability

The original contributions presented in the study are included in the article/Supplementary Material, further inquiries can be directed to the corresponding author.
